# Societal stigma and mistreatment in healthcare among gender minority people: a cross-sectional study

**DOI:** 10.1186/s12939-023-01975-7

**Published:** 2023-08-24

**Authors:** Kristen D. Clark, Mitchell R. Lunn, Jordon D. Bosse, Jae M. Sevelius, Carol Dawson-Rose, Sandra J. Weiss, Micah E. Lubensky, Juno Obedin-Maliver, Annesa Flentje

**Affiliations:** 1 Department of Medical Sciences, Psychiatry, Uppsala University, Uppsala, Sweden; 2 The PRIDE Study/PRIDEnet, Stanford University School of Medicine, 300 Pasteur Drive, Stanford, CA USA; 3 Division of Nephrology, Department of Medicine, Stanford University School of Medicine, 300 Pasteur Drive, Stanford, CA USA; 4 Department of Epidemiology and Population Health, Stanford University School of Medicine, 300 Pasteur Drive, Stanford, CA USA; 5 School of Nursing, Bouvé College of Health Sciences, Northeastern University, 360 Huntington Ave, Boston, MA USA; 6 Center for AIDS Prevention Studies, Department of Medicine, University of California, San Francisco, 513 Parnassus Avenue, San Francisco, CA USA; 7 Center of Excellence for Transgender Health, Department of Medicine, University of California, 513 Parnassus Avenue, San Francisco, San Francisco, CA USA; 8 Department of Community Health Systems, School of Nursing, University of California San Francisco, 2 Koret Way, San Francisco, CA USA; 9 Department of Community Health Systems, UCSF Depression Center, University of California, San Francisco, 2 Koret Way, San Francisco, CA USA; 10 Department of Obstetrics and Gynecology, Stanford University School of Medicine, 300 Pasteur Drive, Stanford, CA USA; 11 Alliance Health Project, Department of Psychiatry, School of Medicine, University of California, San Francisco, 1930 Market Street, San Francisco, CA USA

**Keywords:** Gender minority, Stigma, Discrimination, Healthcare access, Health disparity

## Abstract

**Background:**

Gender minority (GM; individuals whose gender is not aligned with that traditionally associated with the sex that was assigned to them at birth) people have widely reported mistreatment in healthcare settings. Mistreatment is enacted by individuals within society who hold stigmatizing beliefs. However, the relationship between healthcare mistreatment and societal stigma (i.e., the degree to which society disapproves of GM people) is unclear and not measured consistently.

**Methods:**

We analyzed data from 2,031 GM participants in The Population Research in Identity and Disparities for Equality (PRIDE) Study’s 2019 Annual Questionnaire to determine whether societal stigma was associated with participants’ past-year reports of mistreatment (defined as denial of healthcare services and/or lower quality care) in medical or mental healthcare settings. We created a proxy measure of societal stigma by incorporating variables validated in existing literature. Participants reported whether they had experienced mistreatment in medical and mental health settings independently.

**Results:**

Healthcare denial and/or lower quality care during the past year was reported by 18.8% of our sample for medical settings and 12.5% for mental health settings. We found no associations between the societal stigma variables and past-year reports of healthcare denial and/or lower quality care in medical or mental healthcare settings.

**Conclusions:**

Although a high proportion of GM people reported past-year healthcare mistreatment in both medical and mental health settings, mistreatment had no relationship with societal stigma. Factors other than societal stigma may be more important predictors of healthcare mistreatment, such as healthcare workers’ knowledge of and attitudes toward GM people. However, other measures of societal stigma, or different types of mistreatment, may show stronger associations. Identifying key factors that contribute to mistreatment can serve as targets for intervention in communities and healthcare settings.

## Background

Gender minority (GM; individuals whose gender is not aligned with that traditionally associated with the sex assigned to them at birth) people report experiencing a broad range of discriminatory practices in healthcare settings. These experiences include: deficits in healthcare provider knowledge about the unique health needs of GM people, denial of healthcare services, verbal harassment, and physical violence [1–4]. Similar patterns have been identified in mental health settings [3, 5]. Lack of provider competence in caring for GM people and direct mistreatment can have deleterious effects on health and healthcare access, including avoidance of necessary healthcare services [6–10]. Avoidance of healthcare services among GM people has been associated with negative health outcomes such as poor self-reported general health, substance use [11] and increased risk of seeking gender-affirming treatments (e.g., hormone replacement therapy) from outside of the traditional healthcare system, such as from illicit sources [2, 12]. While the risks posed by healthcare mistreatment have been broadly examined, the association of healthcare mistreatment and societal stigma, or the degree to which society disapproves of GM people [13], is less understood.

**Fig. 1:**
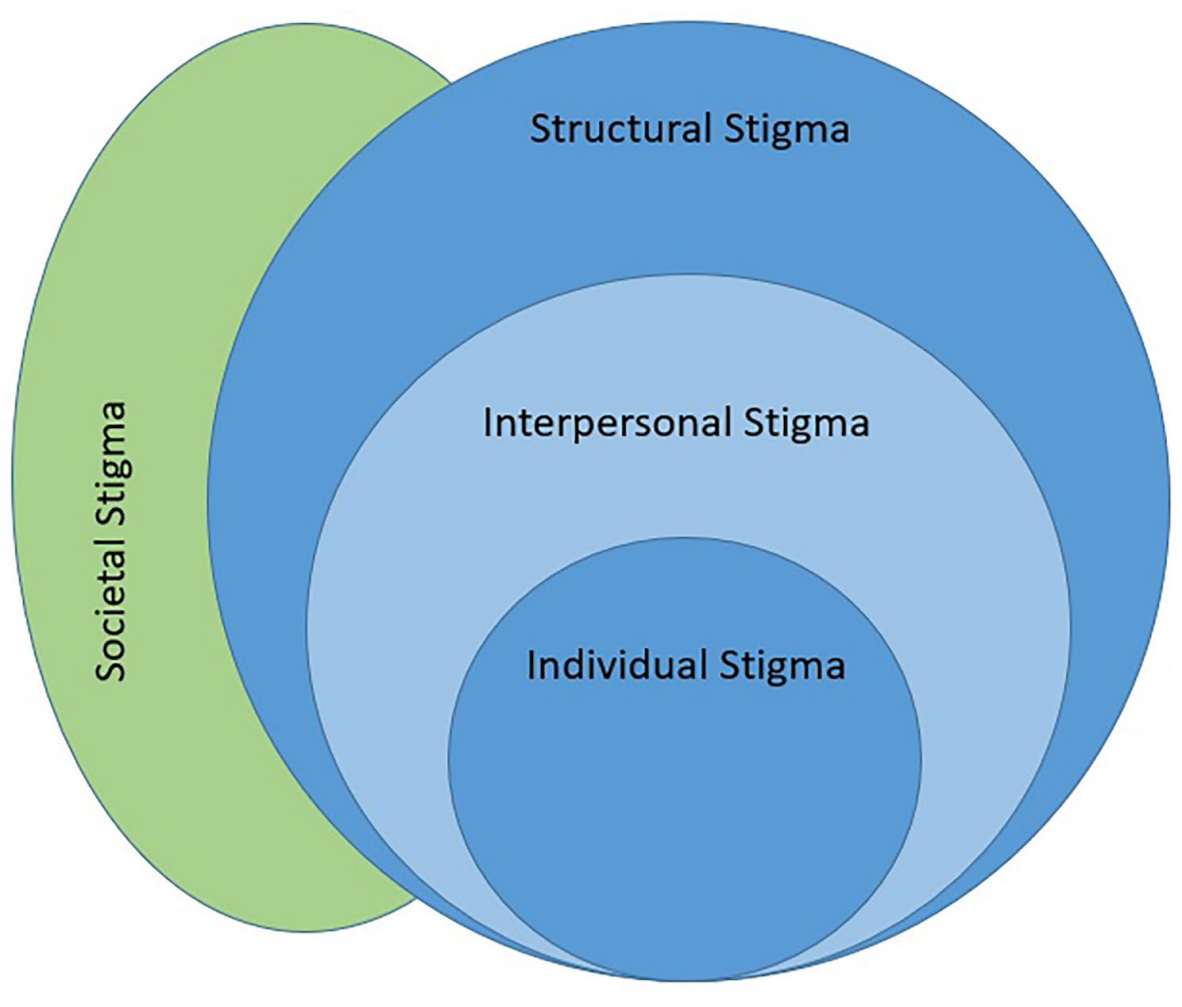
Societal Stigma and Stigma Conceptual Model (adapted from Hughto White et al., 2015)

While structural stigma has been defined as “societal norms and institutional policies that constrain access to resources” [14], we propose that this is actually two constructs: structural and societal stigma. Structural stigma is “institutional policies that constrain access to resources” [14]. Societal stigma, or the degree to which society disapproves of GM people, is determined by cultural norms and standards [13, 15]. Another stigma – interpersonal stigma – relates to direct forms of stigma such as harassment and violence. Whether the broader, dominant society accepts or denies the existence and rights of a marginalized group dictates the policies or laws, their implementation and enforcement, resources alotted to the marginalized group, and individual behaviors deemed socially permissable. Measurement of societal stigma can be viewed as a reflection of other stigma constructs described by Link & Phelan [16]. This relationship is reciprocal; underlying attitudes toward GM people can influence structural, interpersonal, and individual stigma, and they can also influence societal stigma (Fig. 1).

Objective measures of societal stigma, or negative societal attitudes toward GM people, remain absent in scientific literature. Proxy measures of societal stigma in the United States (US) may include state-level policies [17], but the use of summed sexual and gender minority-related (SGM; people whose sexual orientation is not heterosexual and/or individuals whose gender is not aligned with that traditionally associated with the sex assigned to them at birth) state-level policies [18, 19] may be a more accurate reflection of a state’s societal stigma since the action to pass a number of protective policies may indicate the prioritization of GM inclusivity at the state-level. Another US state-level proxy for societal stigma may be the percent of a state that voted for the Republican party candidate in a recent presidential election [20], where greater percentages were associated with greater reports of healthcare services denial among GM participants. These variables may be applied to represent the attitudes toward GM people in a given area. These proxy measures of societal stigma have been associated with a range of outcomes such as poor mental health [17] and greater reports of healthcare services denial [20]. However, little is known about the association between societal stigma and specific forms of interpersonal stigma, such as healthcare mistreatment, defined here as denial of healthcare services or provision of inequitable care. A stronger understanding of this relationship could inform changes in healthcare system to increase equitable healthcare delivery.

## Methods

The purpose of our study was to compare the relationship between selected proxy measures of societal stigma and past-year healthcare mistreatment (i.e., healthcare service denial and/or lower quality care) in medical or mental healthcare settings in a diverse national sample of GM adults in the US. Proxy measures that reflect multiple types of enacted social attitudes were selected for these analyses. We hypothesized that at least one proxy variable for societal stigma (i.e., lower State LGBT + Business Climate Index scores, living in a lower population density area, or lower access to GM inclusive healthcare) would be associated with healthcare service denial and/or lower quality care in medical or mental healthcare settings.

Data were collected within the 2019 Annual Questionnaire of The Population Research in Identity and Disparities for Equality (PRIDE) Study, a national, longitudinal cohort study of sexual and gender minority people who reside in the US. Please see Lunn et al. for detailed description of The PRIDE Study [21]. Briefly, The PRIDE Study is a community-engaged research study with an active Participant Advisory Committee that reviewed and informed the adaptations of measures used in the survey to be inclusive of SGM communities. This committee reviewed and approved the study described here. An extensive recruitment effort for The PRIDE Study included PRIDEnet partners (community partnerships and stakeholders), online communications (e.g., blog posts, newsletters, advertising on social media), in-person outreach at conferences and events, the distribution of The PRIDE Study promotional items, and word-of-mouth. Eligible participants included individuals who were 18 years and older, resided in the US or its territories, self-identified as LGBTQ + or as a sexual and/or gender minority person, and who took 2019 Annual Questionnaire measures outlined in these analyses between June 2019 and May 2020. The GM sample was retained for these analyses, meaning participants who endorsed a gender identity that aligned with the sex that they were assigned at birth were dropped (*n* = 3,629).

### Measures

#### Demographics

Demographics queried of participants included age, race/ethnicity, sexual orientation, gender identity, highest level of education completed, and individual gross income. Age was calculated by subtracting participants’ birth date, obtained upon study enrollment, from the date that the survey was first accessed. Race/ethnicity were measured with a categorical variable (select all that apply). Participants reported sexual orientation with a select-all-that-apply approach that offered 10 options and an open text entry if their preferred label was not listed. Participants reported gender identity with a select-all-that-apply approach that offered 10 options and an open text entry to enter their preferred gender identity if their preferred label was not listed. Highest level of education was measured by an ordinal variable with 10 options ranging from “no schooling” to “Professional degree,” which we then coded as a 4-level variable (i.e., “no high school diploma,” “high school/GED graduate or some college,” “college degree [2- or 4-year],” and “graduate degree”). Individual income was measured by an ordinal 11-item variable ranging from $0 to >$100,000 (collapsed in Table 1). Differences in the experiences of stigma and health vary among different gender identities; therefore, participants were divided into three study population categories to capture the unique experiences [22–24]. Participants who described their gender as non-binary or “another gender identity not listed” were combined in a gender-expansive category. Participants who described their gender identity as transgender man or transgender woman remained in those two separate categories.

**Table 1:** Characteristics of The PRIDE Study 2019 Annual Questionnaire participants

Variable	Total Sample	Gender-expansive People	Transgender Men	Transgender Women
	*(N* = 2,031)	(*n* = 1,119)	(*n* = 626)	(*n* = 280)
*Personal characteristics*				
Age, in years (Mean ± SD)	32.0 (12.2)	30.4 (10.5)	30.0 (10.9)	42.6 (14.7)
Race/ethnicity^a^				
American Indian or Alaska Native	7 (0.4)	3 (0.3)	1 (0.2)	3 (1.1)
Asian	33 (1.7)	24 (2.2)	8 (1.3)	1 (0.4)
Black, African American, or African	30 (1.5)	14 (1.3)	12 (1.9)	4 (1.5)
Hispanic, Latino, or Spanish	50 (2.5)	24 (2.2)	19 (3.1)	7 (2.6)
Middle Eastern or North African	6 (0.3)	3 (0.3)	1 (0.2)	2 (0.7)
White	1,836 (91.9)	1,013 (91.8)	571 (92.3)	252 (91.6)
Another race/ethnicity than is listed	35 (1.8)	22 (2.0)	7 (1.1)	6 (2.2)
Sexual orientation^a^				
Asexual	383 (18.9)	260 (23.2)	85 (13.6)	38 (13.6)
Bisexual	685 (33.7)	383 (34.2)	216 (34.5)	86 (30.7)
Gay	342 (16.8)	146 (13.1)	177 (28.3)	15 (5.4)
Lesbian	301 (14.8)	174 (15.6)	4 (0.6)	120 (42.9)
Pansexual	435 (21.4)	242 (21.6)	116 (18.5)	77 (27.5)
Queer	1,136 (55.9)	736 (65.8)	331 (52.9)	69 (24.6)
Questioning	101 (5.0)	51 (4.6)	34 (5.4)	16 (5.7)
Same-gender loving	104 (5.1)	50 (4.5)	41 (6.6)	13 (4.6)
Straight/heterosexual	85 (4.2)	6 (0.5)	65 (10.4)	14 (5.0)
Two-spirit	22 (1.1)	12 (1.1)	2 (0.3)	8 (2.9)
Another sexual orientation	128 (6.3)	86 (7.7)	25 (4.0)	17 (6.1)
*Socioeconomic Characteristics*				
Annual individual income				
<$20K	1,014 (50.1)	570 (50.1)	347 (55.4)	97 (34.6)
$20K to <$40K	422 (20.8)	259 (23.2)	116 (18.5)	47 (16.8)
$40K to <$60K	267 (13.2)	156 (13.9)	41 (14.6)	70 (11.2)
$60K to <$80k	115 (5.7)	62 (5.5)	25 (4.0)	28 (10.0)
≥$80K	207 (10.2)	72 (6.4)	68 (10.9)	67 (23.9)
Educational level				
No high school diploma	16 (0.8)	6 (0.5)	6 (1.0)	4 (1.4)
High school/GED graduate or some college	705 (34.8)	342 (30.6)	270 (43.2)	93 (33.2)
College degree (2- or 4-year)	781 (38.6)	457 (40.8)	207 (33.1)	117 (41.8)
Graduate degree	522 (25.8)	314 (28.1)	142 (22.7)	66 (23.6)

*Notes*: The number of participants in the study group with available data are reported as (*n*) and percent (%) of *n* for each variable.

^a^Category is not mutually exclusive; therefore, percentages may be greater than 100%.

SD = standard deviation

#### Societal stigma

Three variables were used as proxy variables for societal stigma based on findings from previous analyses [15]. These items were matched to participants based on participant-reported Zone Improvement Plan (ZIP) code.

Population density- Participant ZIP code was converted to Rural-Urban Continuum Codes [25], which identified the population density where the participant resided. These codes were recoded to a single dichotomous variable, indicating urban (participant resides in a designated metropolitan county) and non-urban (participant resides in an area that is not designated as a metropolitan county).

State LGBT + Business Climate Index- Out Leadership, an organization aimed at connecting SGM business leaders, releases an annual report that provided an index on how SGM inclusive each state is to inform business leaders, organizations, and policymakers of “the costs created by polices that create minority stress” [26]. The LGBT + Business Climate Index incorporates data from the Movement Advancement Project, the United States Transgender Survey, The Williams Institute, the Bureau of Labor Statistics, and the United States Treasury to create a score for each state that ranges from 25 to 100 from points allotted from five domains: “Legal and Nondiscrimination Protections,” “Youth and Family Support,” “Political and Religious Attitudes,” “Health Access and Safety,” and “Work Environment and Employment.” A sensitivity analysis comparing the variable in continuous and ordinal forms was performed. No statistically meaningful difference was observed. Therefore, these scores were included as a single continuous variable to retain as much information as possible, where higher values indicate a more positive environment for SGM people.

Leaders in LGBTQ Healthcare Equality- The Human Rights Campaign (HRC) provides an annual Healthcare Equality Index report in which hospitals are scored by calculating the number of SGM-inclusive policies they implemented within four domains: “Patient-Centered Care,” “Patient Services and Support,” “Employee Benefits and Policies,” and “Community Engagement” [27]. The total possible score ranges from 0 to 100. A score of 100 gives a healthcare facility the designation of “Leader in LGBTQ Healthcare Equality.” The total estimated GM population in each state [28] was divided by the total number of healthcare facilities with the “Leader in LGBTQ Healthcare Equality” designation to create a continuous variable accounting for different estimated proportions of GM people in each state. A lower number indicates better access to inclusive care, or lower societal stigma as inclusive care is likely to be more valued where there is better access.

Proxy measures of societal stigma that incorporate sexual orientation in addition to gender identity in the development of their variables (i.e., State LGBT + Business Climate Index, Leaders in LGBTQ Healthcare Equality) were retained as developed. This is because the measures did not provide unique data for societal stigma as related to sexual orientation versus gender identity. Additionally, our GM sample (*n* = 2031) is comprised of almost 96% SM. Therefore, measurement of social climate in regard to both sexual orientation and gender identity are important to GM people’s experiences [29].

#### Healthcare service denial and/or lower quality of care

Healthcare mistreatment was measured by two dependent variables. Participants reported whether they had been refused healthcare services or given lower quality (a) medical or (b) mental healthcare in the past 12 months. If they reported ‘yes,’ they were then asked whether they attributed this experience to their gender identity and/or expression. A dichotomous variable was created for each healthcare setting, medical or mental health. Participants who did not access care in the past 12 months were excluded from analysis (*n* = 53).

### Analysis

Descriptive statistics were used for demographic variables and past-year medical and mental healthcare discrimination among the three gender identity categories: gender-expansive people, transgender men, and transgender women. In our analyses, where sexual orientation and race/ethnicity were included as covariates, each of the response options were dichotomously coded to account for multiple selections of race/ethnicity or sexual orientation.

Logistic regression analysis was used to determine the extent to which the three societal stigma variables (i.e., State LGBT + Business Climate Index scores, population density where one lives, and access to GM inclusive healthcare) predicted the odds of past-year healthcare mistreatment. Separate models tested the relationship between each of the three societal stigma variables and the two mistreatment variables (i.e., reported past-year healthcare service denial and/or lower quality care in medical settings and in mental health settings). Age, race/ethnicity, sexual orientation, education level, and income were included in all models as covariates. All models were run for each of the three gender identity categories using Stata 15 [30].

## Results

### Study sample

Participant characteristics are presented in Table 1. A total of 2,031 GM participants were included in these analyses; 55.1% (*n* = 1,119) were gender-expansive people, 30.3% (*n* = 626) were transgender men, and 13.8% (*n* = 280) were transgender women. The mean age of participants was 32.0 years (standard deviation [SD] = 12.2), and the sample was predominantly White (*n* = 1,836; 91.9%). Nearly two-thirds (64.4%, *n* = 1,303) of participants had earned a college degree, and half (50.1%, *n* = 1,014) reported an individual income of less than $20,000 annually. Approximately 89% of our sample lived in a high population density area. The mean State LGBT + Leaders Equality Index score for our sample was 68.99 (SD = 19.08). The mean number of GM people per ‘Leader in LGBTQ Healthcare Equality’-listed hospital was 5,133 (range 871 − 20,892).

### Reported past-year denial or lower quality of medical care

On average, 18.8% (*n* = 378) of GM people reported being denied or given lower quality medical care in the past-year. Within our study groups, 19.4% (*n* = 215) of gender-expansive people, 19.1% (*n* = 118) of transgender men, and 16.3% (*n* = 45) of transgender women reported past-year denial or lower quality medical care. The results of models evaluating societal stigma in relation to reported past-year medical discrimination are presented in Table 2. There was no relationship between societal stigma and reported past-year denial or lower quality medical care among any of the gender identity categories.

**Table 2:** Results of Models Evaluating Societal Stigma in Relation to Reported Past-Year Mistreatment in Medical Settings

	Gender-Expansive			Transgender Men			Transgender Women		
Past-Year Medical Healthcare Mistreatment	aOR	95% CI	*p*	OR	95% CI	*p*	OR	95% CI	*p*
Model 1: Lives in a metropolitan area	1.1	0.62–1.92	0.75	1.17	0.63–2.17	0.629	1.99	0.64–5.51	0.247
Model 2: State LGBT + Business Climate Index	1.01	1.00-1.02	0.106	1.01	1.00-1.02	0.244	0.99	0.97–1.01	0.393
Model 3: Healthcare Equality Index	1	1.00–1.00	0.75	1	1.00–1.00	0.56	1	1.00–1.00	0.499

aOR = adjusted odds ratio

CI = confidence interval

Covariates in analyses included age, sexual orientation, race/ethnicity, individual income, education level, and sex assigned at birth (only for gender-expansive group)

### Reported past-year denial or lower quality of mental healthcare

On average, 12.5% (*n* = 219) of GM people reported being denied or given lower quality mental healthcare in the past-year. Within our study groups, 12.3% (*n* = 118) of gender-expansive people, 14.6% (*n* = 80) of transgender men, and 8.6% (*n* = 21) of transgender women reported past-year mental healthcare denial or lower quality care. The results of models evaluating societal stigma in relation to reported past-year mental healthcare denial or lower quality care are presented in Table 3. There was no relationship between societal stigma and reported past-year mental healthcare denial or lower quality care reported by any of the gender identity categories.

**Table 3:** Results of Models Evaluating Societal Stigma in Relation to Reported Past-Year Healthcare Mistreatment in Mental Healthcare Settings

	Gender-Expansive			Transgender Men			Transgender Women		
Past-Year Mental Healthcare Mistreatment	aOR	95% CI	*p*	OR	95% CI	*p*	OR	95% CI	*p*
Model 1: Lives in a metropolitan area	0.67	0.29–1.54	0.34	1.54	0.72–3.28	0.259	0.34	0.03–3.45	0.361
Model 2: State LGBT + Business Climate Index	1.01	1.00-1.02	0.09	1.01	0.99–1.02	0.281	1	0.97–1.03	0.952
Model 3: Healthcare Equality Index	1	1.00–1.00	0.429	1	1.00–1.00	0.172	1	1.00–1.00	0.454

aOR = adjusted odds ratio

CI = confidence interval

Covariates in analyses included age, sexual orientation, race/ethnicity, individual income, education level

## Discussion

Nearly one-fifth of GM people in our sample reported being denied or given lower quality medical care within the past year, with gender-expansive people (19.4%) and transgender men (19.1%) reporting the highest prevalence. While previous research found that transgender men report higher levels of healthcare mistreatment compared to transgender women [6], our findings revealed gender-expansive people are also vulnerable. Although there have been increased efforts to provide gender-affirming services in recent years, these efforts have been focused on supporting a binary construct of gender where medical care enhances gender expression consistent with stereotypical presentation as either man or woman [31, 32]. Transgender women in particular have historically been the focus of healthcare research, which may be why the prevalence of denial or lower quality medical care in this group is somewhat lower, as most healthcare system interventions have been with this group in mind [2]. Further, our sample was predominantly white; we know that GM people of color experience discrimination and other types of mistreatment at greater rates [33–35]. Our sample is highly educated although with relatively low annual income compared to more representative samples of GM people [36]. Having greater economic resources implies that participants may have more choices in what providers they see; however, given the lower income of our sample, there may be variations in the types of insurance to which they may have access, such as public sources (e.g., Medicaid) or private insurance; this may impact our findings. People with public insurance in the US have fewer choices, even more so when seeking providers with competency in GM healthcare needs or for accessing gender-affirming interventions [37]. Providers’ lack of awareness of gender-expansive people as a gender minority group and that they may desire gender-affirming interventions can create significant barriers to care engagement [38].

Further, we found that 12.5% of our sample reported denial or lower quality of mental healthcare within the past year with transgender men (14.6%) reporting the highest prevalence, followed by gender-expansive people (12.3%), and transgender women (8.6%). No other study to date, to our knowledge, has independently examined the prevalence of healthcare mistreatment among GM people in mental health settings. Limited available evidence suggests GM people experience enacted stigma, such as discrimination, and in obtaining mental health services [39]. Yet GM people are known to have a high prevalence of depression, suicide, and other mental health disparities [40, 41] and, therefore, are likely to require access to mental health services. Further work to evaluate GM individuals’ experiences in mental health treatment, particularly inpatient psychiatric settings where crisis stabilization treatment is provided, and how mental settings can improve care for GM people is critical to addressing these needs and improving long-term mental health outcomes.

Contrary to our hypotheses, there were no relationships between any variables representing societal stigma and the reports of denial or lower quality care in medical or mental healthcare settings among GM participants. This could be due to the lack of sensitivity and/or specificity our proxy measures of societal stigma. Other forms of stigma, such as individual (self directed stigma or anticipation of stigma) or interpersonal stigma (experiences of stigma), not reflected in our analyses could be better indicators of societal stigma. Our study tested the measures found to be most promising based on previous analyses [15] and the availability of SGM-inclusive healthcare in a state, but we did not find an association between these markers of societal stigma and being denied or given lower quality healthcare. Given our null findings, what could be important is to examine the relationship between societal stigma and efforts to educate healthcare workers to provide inclusive care to GM people. For example, when interventions to improve the abilty of healthcare workers to provide competent care to GM are utilized in a community where negative attitudes toward GM people are prevalent, this could result in less effectiveness as opposed to the same intervention applied in a community where attitudes are more positive. Further, societal stigma could be a predictor of unconscious bias as broader community attitudes could normalize and reinforce stigmatizing behaviors and interactions that may be unintentional. However, currently available variables representing societal stigma are quite imperfect and will require refinement and further research to better define and measure this construct. The role of societal stigma as in efforts to create inclusive health education should be explored. Efforts to improve the education of practicing clinicians (e.g., nurses, physicians) on the healthcare needs of GM people have been made in at least some healthcare facilities [42, 43] and in clinical education [44–49]. However, the effects of these efforts may not be enough to counteract the stigmatizing beliefs surrounding gender non-conformity or to consistently combat systemic transphobia. In one study, transphobia (a form of societal stigma) was a stronger predictor of mistreatment of GM people in healthcare settings than cultural competency education [50], indicating that societal stigma could contextualize where there may be variations in the effectiveness of cultural competency education. For example, communities with greater transphobia may be slower to enact cultural competency education and when enacted, it may take longer to influence delivery of patient care. Therefore, development of a measure of community level societal stigma could be a promising representation of the phenomenon [51]. Previous research suggests that lifetime experiences of healthcare discrimination or treatment denial can result in future avoidance or delay of accessing necessary healthcare services [19, 24, 52]. Similarly, structural barriers in the US such as access to health insurance, whether an individual’s policy covers gender affirming treatment, or long wait times may also be impediments to seeking healthcare [53–55]. However, structural barriers to mental health services among GM people are understudied [56].

Measurement of healthcare mistreatment in the broader literature on GM people’s experiences is another consideration of our findings. Dichotomous answer options to indicate mistreatment in medical or mental health settings limit the variability in GM people’s reports of their experiences. This is also observed in other studies where whether healthcare was accessed as a yes/no responses and the same for experience discrimination or other types of mistreatment [6, 57, 58]. Some studies evaluated different types of mistreatment in healthcare, but those responses are also reduced to yes/no with little specificity of where the mistreatment occurred, how often, clinicians/support staff involved, or other details that could help guide interventions. Similar barriers were observed in our study where mistreatment was a single question that was asking about two different experiences, “denial or lower quality of care.” A participant who experienced lower quality of care one time in the past year was represented equally with a person who may have experienced both denial and lower quality care many times across the past year. The question did not define lower quality of care, potentially leading to measurement error.

### Strengths and limitations

This study discussed the importance of measuring societal stigma distinct from other contructs of stigma and evaluated proxy measures for the construct. We established a prevalence for past-year denial or low-quality care in mental health settings, rarely discussed separately from other types of healthcare mistreatment in the broader literature. We also tested the use of the State LGBT + Business Index as a potential indicator of stigma exposure among GM; no studies, to our knowledge, have evaluated its use in this capacity. Our sample is substantial and represents diverse gender identities across the US, allowing us to perform important subgroup analyses of these distinct gender identitity groups. Despite the importance of this study, limitations remain. The cross-sectional study design limited our ability to determine causality; therefore, our results were correlational. Future work involving longitudal or prospective approaches is an important consideration to advance our understanding of societal stigma and healthcare mistreatment as these differences may be more evident over time as opposed to a singular cross-sectional analysis. Similarly, expanding measurement of societal stigma to be representative of global communities is an important consideration. Some work has looked at country level policies but few look at more localized measures and how those relate to community attitudes [59]. Further, two of our proxies for societal stigma (State LGBT + Business Climate Index, Leaders in LGBTQ Healthcare Equality) included elements that reflected state societal stigma related to sexual orientation and gender identity. While this is relevant to our highly sexually diverse sample (almost 96% sexual minority), future work should examine whether a GM-specific measure of societal stigma could be an improvement. This would particularly be important in a sample with more straight/heterosexual GM participants and to uniquely capture the issues faced by GM people not faced by cisgender SM people. The Leaders in LGBTQ Healthcare Equality variable poses a limitation as it represents LGBTQ inclusivity within inpatient hospital settings. It does not represent the availability of inclusive and affirming outpatient care, which could be disproportionate to the number of hospitals given this designation (e.g., few hospitals may have the designation, but there may be more GM-inclusive outpatient resources and on the whole more healthcare for LGBTQ + people may occur in outpatient sites). Sample recruitment relied on convenience sampling; therefore, our sample was not representative of the broader GM population. Most specifically, we had a high proportion of white participants who were highly educated with relatively high income, which is considerably different than estimations of the broader GM population [6, 60]. GM people of color face higher rates of discrimination within and outside of healthcare [2], thus the sample composition likely impacted our results. Our sample was composed of GM adults, who may have had different experiences in accessing medical or mental healthcare than GM young adults or youth. Similarly, our sample lived predominantly in higher population density areas, leading to less granularity in our analyses to differentiate experiences of GM people in rural settings. Therefore, our findings were not generalizable to those populations. Further, any measure of societal stigma should be tested among a diverse sample due to the multiple forms of oppression experienced by individuals with multiple minoritized identities (e.g., one who is GM and sexual minority, one who is GM and Black; GM people who are immigrants or refugees [56, 57]. The focus on mistreatment in medical and mental health settings broadly may obscure understanding of differences between care environments in which GM individuals have experienced healthcare mistreatment such as medical laboratories or pharmacies in contrast to healthcare provider offices [38]. Further work asking about experiences in specific healthcare settings may address this gap and form targeted opportunities for intervention and evaluation.

#### Future directions

Future studies should explore the use of electronic health records to evaluate the care GM patients receive for specific conditions and allow for a nuanced comparison with cisgender patients in combination with self-report measures. Continued improvement is needed in the measurement of societal stigma. Our results suggest that societal stigma is not associated with reported healthcare service denial or lower quality of care of GM people; but this could be due to the specific measures used to assess societal stigma and denial/quality of care. For example, the State LGBT + Business Climate Index is composed of items that measure structural conditions *(e.g.*., Legal and Nondiscrimination Protections) and societal stigma (e.g., political and religious attitudes). Other existing measures of attitudes toward transgender people cannot be disaggregated by state. As such, routine collection of individuals’ attitudes toward GM people in large, nationally representative samples could help us measure societal attitudes directly. The ability to identify state of residence of respondents in association with attitudes is important. Societal attitudes, positive or negative, may be enacted through state-level policies. General attitudes in a particular area could shift, but changes to state-level policies could lag. Examples of this have been observed such as when the “bathroom bills” (i.e., legislation requiring people to use bathrooms as designated based on one’s sex assigned at birth) were a focus of several state’s legislative sessions yet were unpopular with the US public [61].

## Conclusions

GM people continue to experience both denial and lower quality of care in medical and mental healthcare settings. Despite less societal stigma in some areas, no difference was observed using the potential measures of societal stigma. Mistreatment in mental health settings is an important area for focused research among GM people who have considerable disparities in mental health outcomes. Further research that employs more nuanced and detailed measures of mistreatment in healthcare broadly is needed to improve the healthcare experiences of GM people, and these relationships should be evaluated in diverse GM study populations globally and in communities.

## Data Availability

The PRIDE Study is a community-based research project. LGBTQ + populations have historically been marginalized, which has impacted the trust between communities and institutions. Therefore, part of the guidelines of The PRIDE Study is restricted access to the participants’ data. Access to the data can be requested by submitting an ancillary study application at https://pridestudy.org/ and following the application process.

## Abbreviations

GM: Gender minority
LGBT+: Lesbian, gay, bisexual, transgender, and other related sexual orientations and gender identities that are not heterosexual or cisgender (i.e., one whose gender is the same as that which is traditionally associated with their sex assigned at birth).

## References

[1] Ayhan CHB, Bilgin H, Uluman OT, Sukut O, Yilmaz S, Buzlu S (2019). A systematic review of the discrimination against sexual and gender minority in Health Care settings. Int J Health Serv.

[2] Cicero EC, Reisner SL, Silva SG, Merwin EI, Humphreys JC (2019). Health Care Experiences of Transgender adults: an Integrated mixed Research Literature Review. Adv Nurs Sci.

[3] Zeeman L, Sherriff N, Browne K, McGlynn N, Mirandola M, Gios L (2019). A review of lesbian, gay, bisexual, trans and intersex (LGBTI) health and healthcare inequalities. Eur J Public Health.

[4] Markovic L, McDermott DT, Stefanac S, Seiler-Ramadas R, Iabloncsik D, Smith L (2021). Experiences and interactions with the healthcare system in transgender and non-binary patients in Austria: an exploratory cross-sectional study. Int J Environ Res Public Health.

[5] Snow A, Cerel J, Loeffler DN, Flaherty C (2019). Barriers to mental health care for transgender and gender-nonconforming adults: a systematic literature review. Health Soc Work.

[6] James S, Herman J, Rankin S, Keisling M, Mottet L, Anafi M. The report of the 2015 US transgender survey. 2016.

[7] Kcomt L (2019). Profound health-care discrimination experienced by transgender people: rapid systematic review. Soc Work Health Care.

[8] Koehler A, Motmans J, Mulió Alvarez L, Azul D, Badalyan K, Basar K et al. How the COVID-19 pandemic affects transgender health care - A cross-sectional online survey in 63 upper-middle-income and high-income countries. Int J Transgender Health. 2021;1–14. 10.1080/26895269.2021.1986191 PMC10373616 37519919

[9] Socías ME, Marshall BD, Arístegui I, Romero M, Cahn P, Kerr T (2014). Factors associated with healthcare avoidance among transgender women in Argentina. Int J Equity Health.

[10] Leite BO, de Medeiros DS, Magno L, Bastos FI, Coutinho C, de Brito AM (2021). Association between gender-based discrimination and medical visits and HIV testing in a large sample of transgender women in northeast Brazil. Int J Equity Health.

[11] Reisner SL, Pardo ST, Gamarel KE, Hughto JMW, Pardee DJ, Keo-Meier CL (2015). Substance use to cope with Stigma in Healthcare among U.S. female-to-male trans masculine adults. LGBT Health.

[12] Glick JL, Andrinopoulos KM, Theall KP, Kendall C (2018). Tiptoeing around the System”: Alternative Healthcare Navigation among gender minorities in New Orleans. Transgender Health.

[13] Hasenbush A, Flores A, Kastanis A, Sears B, Gates G. The LGBT divide: A data portrait of LGBT people in the Midwestern, Mountain & Southern states. 2014.

[14] White Hughto JM, Reisner SL, Pachankis JE (2015). Transgender stigma and health: a critical review of stigma determinants, mechanisms, and interventions. Soc Sci Med.

[15] Clark K. Measures of Societal Stigma and Their Association to Components of Minority Stress Among Gender Minority People. In: NURSING RESEARCH. LIPPINCOTT WILLIAMS & WILKINS TWO COMMERCE SQ, 2001 MARKET ST, PHILADELPHIA … p. S58–S58.

[16] Link BG, Phelan JC (2001). Conceptualizing Stigma. Annu Rev Sociol.

[17] Hatzenbuehler ML, Keyes KM, Hasin DS (2009). State-Level Policies and Psychiatric Morbidity in Lesbian, Gay, and bisexual populations. Am J Public Health.

[18] Clark K, Lunn M, Lev E, Trujillo M, Lubensky M, Capriotti M et al. Policy protections and their relationship to discrimination and victimization among sexual and gender minority people. In: APHA’s 2020 Virtual Annual Meeting and Expo, October. 2020. p. 24–8.

[19] Clark KD, Luong S, Lunn MR, Flowers E, Bahalkeh E, Lubensky ME et al. Healthcare Mistreatment, State-Level Policy Protections, and Healthcare Avoidance among gender minority people. Sex Res Social Policy. in press. 10.1007/s13178-022-00748-1 PMC9701649 36458212

[20] White Hughto JM, Murchison GR, Clark K, Pachankis JE, Reisner SL (2016). Geographic and Individual differences in Healthcare Access for U.S. Transgender adults: a Multilevel Analysis. LGBT Health.

[21] Lunn MR, Lubensky M, Hunt C, Flentje A, Capriotti MR, Sooksaman C (2019). A digital health research platform for community engagement, recruitment, and retention of sexual and gender minority adults in a national longitudinal cohort study–the PRIDE study. J Am Med Inform Assoc JAMIA.

[22] Cicero EC, Reisner SL, Merwin EI, Humphreys JC, Silva SG (2020). The health status of transgender and gender nonbinary adults in the United States. PLoS ONE.

[23] Kattari SK, Atteberry-Ash B, Kinney MK, Walls NE, Kattari L (2019). One size does not fit all: differential transgender health experiences. Soc Work Health Care.

[24] Kattari SK, Bakko M, Langenderfer-Magruder L, Holloway BT. Transgender and nonbinary experiences of victimization in health care. J Interpers Violence. 2020;0886260520905091. 10.1177/0886260520905091 32046594

[25] United States Department of Agriculture.,. Rural-Urban Continuum Codes [Internet]. United States Department of Agriculture,. [cited 2021 Mar 1]. Available from: https://www.ers.usda.gov/data-products/rural-urban-continuum-codes.aspx.

[26] OUT Leadership,. State LGBT + Business Climate Index [Internet]. OUT Leadership. ; 2019. Available from: https://outleadership.com/wp-content//uploads/2019/05/StateClimateIndex_May1_FINAL_digital.pdf.

[27] Human Rights Campaign.,. Healthcare Equality Index 2019 [Internet]. 2019 [cited 2021 Mar 14]. Available from: https://www.thehrcfoundation.org/professional-resources/leaders-in-lgbt-healthcare-equality.

[28] Herman JL, Flores AR, Brown, Taylor NT, Wilson, Bianca DM. Age of individuals who identify as transgender in the United States [Internet]. The Williams Institute and UCLA Center for Health Policy Research; 2017 [cited 2021 Mar 28]. Available from: https://williamsinstitute.law.ucla.edu/wp-content/uploads/Age-Trans-Individuals-Jan-2017.pdf.

[29] Clark KD, Lunn MR, Lev EM, Trujillo MA, Lubensky ME, Capriotti MR et al. State-Level Policy environments, discrimination, and victimization among sexual and gender minority people. Int J Environ Res Public Health. 2022;19(16). 10.3390/ijerph19169916 PMC9407724 36011548

[30] Stata Corp (2017). Stata Statistical Software: release 15. College Station.

[31] Lykens JE, LeBlanc AJ, Bockting WO (2018). Healthcare experiences among young adults who identify as genderqueer or nonbinary. LGBT Health.

[32] Paine EA (2018). Embodied disruption:“Sorting out” gender and nonconformity in the doctor’s office. Soc Sci Med.

[33] Howard SD, Lee KL, Nathan AG, Wenger HC, Chin MH, Cook SC (2019). Healthcare Experiences of Transgender People of Color. J Gen Intern Med.

[34] Kattari SK, Walls NE, Whitfield DL, Langenderfer-Magruder L (2015). Racial and ethnic differences in experiences of discrimination in Accessing Health Services among Transgender People in the United States. Int J Transgenderism.

[35] Seelman KL, Vasi A, Kattari SK, Alvarez-Hernandez LR (2021). Predictors of healthcare mistreatment among transgender and gender diverse individuals: are there different patterns by patient race and ethnicity?. Soc Work Health Care.

[36] Meyer IH, Brown TN, Herman JL, Reisner SL, Bockting WO (2017). Demographic characteristics and health status of transgender adults in select US regions: behavioral risk factor Surveillance System, 2014. Am J Public Health.

[37] Mallory C, Tentindo W. Medicaid Coverage for Gender-Affirming Care [Internet]. WIlliams Institute, UCLA School of Law; 2022 [cited 2022 Dec 22]. Available from: https://williamsinstitute.law.ucla.edu/wp-content/uploads/Medicaid-Gender-Care-Dec-2022.pdf.

[38] Puckett JA, Cleary P, Rossman K, Newcomb ME, Mustanski B. Barriers to Gender-Affirming Care for Transgender and Gender Nonconforming Individuals. Sex Res Soc Policy J NSRC SR SP. 2017/08/04 ed. 2018;15(1):48–59. 10.1007/s13178-017-0295-8 PMC5842950 29527241

[39] White BP, Fontenot HB. Transgender and non-conforming persons’ mental healthcare experiences: an integrative review. Arch Psychiatr Nurs. 2019. 10.1016/j.apnu.2019.01.005 30927991

[40] Valentine SE, Shipherd JC (2018). A systematic review of social stress and mental health among transgender and gender non-conforming people in the United States. Clin Psychol Rev.

[41] Blondeel K, De Vasconcelos S, García-Moreno C, Stephenson R, Temmerman M, Toskin I (2018). Violence motivated by perception of sexual orientation and gender identity: a systematic review. Bull World Health Organ.

[42] Klein EW, Nakhai M (2016). Caring for LGBTQ patients: methods for improving physician cultural competence. Int J Psychiatry Med.

[43] Wyckoff ED (2019). LGBT cultural competence of acute care nurses. J Nurses Prof Dev.

[44] Cooper MB, Chacko M, Christner J. Incorporating LGBT health in an undergraduate medical education curriculum through the construct of social determinants of health. MedEdPORTAL. 2018;14. 10.15766/mep_2374-8265.10781 PMC6342423 30800981

[45] Kelley L, Chou CL, Dibble SL, Robertson PA (2008). A critical intervention in lesbian, gay, bisexual, and transgender health: knowledge and attitude outcomes among second-year medical students. Teach Learn Med.

[46] Klotzbaugh RJ, Ballout S, Spencer G (2020). Results and implications from a gender minority health education module for advance practice nursing students. J Am Assoc Nurse Pract.

[47] Mayfield JJ, Ball EM, Tillery KA, Crandall C, Dexter J, Winer JM et al. Beyond men, women, or both: a comprehensive, LGBTQ-inclusive, implicit-bias-aware, standardized-patient-based sexual history taking curriculum. MedEdPORTAL. 2017;13. 10.15766/mep_2374-8265.10634 PMC6338175 30800835

[48] Sherman AD, McDowell A, Clark KD, Balthazar M, Klepper M, Bower K (2020). Transgender and gender diverse health education for future nurses: students’ knowledge and attitudes. Nurse Educ Today.

[49] Walsh D, Hendrickson SG (2015). Focusing on the “T” in LGBT: an online survey of related content in Texas nursing programs. J Nurs Educ.

[50] Stroumsa D, Shires DA, Richardson CR, Jaffee KD, Woodford MR (2019). Transphobia rather than education predicts provider knowledge of transgender health care. Med Educ.

[51] Michaels EK, Board C, Mujahid MS, Riddell CA, Chae DH, Johnson RC et al. Area-level racial prejudice and health: a systematic review. Health Psychol. 2022. 10.1037/hea0001141 PMC8930473 35254858

[52] Kcomt L, Gorey KM, Barrett BJ, McCabe SE (2020). Healthcare avoidance due to anticipated discrimination among transgender people: a call to create trans-affirmative environments. SSM - Popul Health.

[53] Bakko M, Kattari SK. Transgender-related insurance denials as barriers to Transgender Healthcare: differences in experience by insurance type. J Gen Intern Med. 2020;1–8. 10.1007/s11606-020-05724-2 PMC7280420 32128693

[54] Clark KD, Sherman AD, Flentje A. Health insurance prevalence among gender minority people: a systematic review and meta-analysis. Transgender Health. 2021. 10.1089/trgh.2020.0182 PMC9398476 36033215

[55] Macapagal K, Bhatia R, Greene GJ (2016). Differences in healthcare access, use, and experiences within a community sample of racially diverse lesbian, gay, bisexual, transgender, and questioning emerging adults. LGBT Health.

[56] Clark KD, Capriotti MR, Obedin-Maliver J, Lunn MR, Lubensky ME, Flentje A. Supporting sexual and gender minority health: research priorities from mental health professionals. J Gay Lesbian Ment Health. 2019;1–17.

[57] Grant JM, Motter LA, Tanis J. Injustice at every turn: A report of the National Transgender Discrimination Survey [Internet]. Washington, D.C.: National Center for Transgender Equality and National Gay and Lesbian Task Force; 2011 [cited 2020 Jul 17]. Available from: http://dataspace.princeton.edu/jspui/handle/88435/dsp014j03d232p.

[58] Narayan A, Lebron-Zapata L, Morris E (2017). Breast cancer screening in transgender patients: findings from the 2014 BRFSS survey. Breast Cancer Res Treat.

[59] Bränström R, Pachankis JE. Country-level structural stigma, identity concealment, and day-to-day discrimination as determinants of transgender people’s life satisfaction. Soc Psychiatry Psychiatr Epidemiol [Internet]. 2021; Available from: 10.1007/s00127-021-02036-6. 10.1007/s00127-021-02036-6 PMC8429389 33582826

[60] Carpenter CS, Eppink ST, Gonzales G (2020). Transgender Status, gender identity, and socioeconomic outcomes in the United States. ILR Rev.

[61] Jones RP, Cox D, Cooper B, Lienesch R. Majority of Americans Oppose Transgender Bathroom Restrictions [Internet]. PRRI; 2017 Mar. Available from: http://www.prri.org/research/lgbt-transgender-bathroom-discrimination-religious-liberty/.

